# Biliary NF‐κB‐Inducing Kinase Promotes Ductular Reaction, Inflammation, and Fibrosis and Impedes Liver Disease Resolution

**DOI:** 10.1096/fj.202502014RRR

**Published:** 2025-12-05

**Authors:** Yina Wang, Rohollah Nikooie, Qianqian Kang, Liangyou Rui

**Affiliations:** ^1^ Department of Molecular and Integrative Physiology University of Michigan Medical School Ann Arbor Michigan USA; ^2^ Department of Exercise Physiology, Faculty of Physical Education and Sport Sciences Shahid Bahonar University of Kerman Kerman Iran; ^3^ Division of Gastroenterology and Hepatology, Department of Internal Medicine University of Michigan Medical School Ann Arbor Michigan USA; ^4^ Elizabeth Weiser Caswell Diabetes Institute University of Michigan Ann Arbor Michigan USA

**Keywords:** cholangiocyte, ductular reaction, fibrosis, inflammation, liver injury, MAP3K14, NF‐κB‐inducing kinase

## Abstract

NF‐κB‐inducing kinase (NIK) is selectively upregulated in cholangiocytes during chronic liver disease, but its role in disease progression is poorly understood. We here show that cholangiocyte‐specific overexpression of NIK (*NIK* ^*Tg*^) exacerbates liver disease progression. *NIK* ^*Tg*^ mice were generated using a CK19‐CreERT/loxp system. *NIK* ^*Tg*^ and wild‐type control (*NIK* ^*Con*^) mice were treated with thioacetamide (TAA) or 3,5‐diethoxycarbonyl‐1,4‐dihydrocollidine (DDC) to induce liver disease. In a separate cohort, mice were allowed a 2‐week recovery period following 4 weeks of TAA administration. NIK was also ablated in human HuCC‐T1 cholangiocyte cultures (HuCC‐T1^NIK−/−^), and either wild‐type NIK or the NIK^G885R^ mutant (defective in activating IKKα) was reintroduced into HuCC‐T1^NIK−/−^ cells. We found that *NIK* ^*Tg*^ mice exhibited a markedly elevated liver pathology—pronounced cholangiocyte expansion, liver inflammation, and fibrosis—upon exposure to DDC or TAA, leading to death. *NIK* ^*Tg*^ mice, unlike *NIK* ^*Con*^ controls, failed to recover after cessation of toxin exposure. Deletion of *NIK* in HuCC‐T1^NIK−/−^ cells reduced cell proliferation and survival and blunted secretion of cholangiokines that activate macrophages and hepatic stellate cells (HSCs). Re‐introduction of NIK into HuCC‐T1^NIK−/−^ cells fully rescued these defects. In contrast, re‐introduction of NIK^G885R^ partially rescued the defects. Collectively, these findings indicate that biliary NIK drives ductular reaction and pathogenic cholangiokine secretion through both IKKα‐dependent and ‐independent mechanisms. Furthermore, aberrant biliary NIK impedes liver injury resolution and fibrosis regression, thereby contributing to poor outcomes.

## Graphical abstract

Pathogenic factors stimulate biliary NIK, which induces aberrant cholangiocyte expansion and ductular reaction, leading to cholestatic injury. In addition, NIK stimulates secretion of cholangiokines that activate macrophages/Kupffer cells and hepatic stellate cells (HSCs), thereby driving liver inflammation and fibrosis and impairing liver injury resolution.

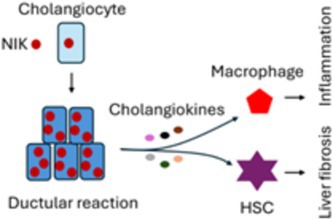

## 1 Introduction

Bile duct epithelial cells, also called cholangiocytes, line the bile ducts where they facilitate bile transport and modify its composition. In chronic liver disease, cholangiocytes become activated and differentiate into reactive cholangiocytes, leading to ductular reaction characterized by cholangiocyte proliferation and expansion [1]. Reactive cholangiocytes acquire new pro‐inflammatory and profibrogenic properties [1, 2, 3]. However, the molecular mechanisms underpinning cholangiocyte reactivation and ductular reaction remain poorly understood.

We recently reported that NF‐κB‐inducing kinase (NIK), also known as MAP3K14, is upregulated selectively in cholangiocytes in both humans and mice with chronic liver disease, particularly cholestatic liver disease [4]. This raises the possibility that aberrant activation of biliary NIK may promote differentiation of reactive cholangiocytes and ductular reaction. NIK is known to activate the noncanonical NF‐κB2 pathway in response to a subset of cytokines [5]. It binds to, phosphorylates, and activates IKKα, and IKKα in turn phosphorylates NF‐κB2 precursor p100 [6]. Phosphorylation triggers the cleavage of p100 into p52, a transcriptionally active transcription factor [5, 7, 8, 9]. The noncanonical NIK/IKKα/NF‐κB2 pathway is known to control the development of the thymus and lymphoid organs and is essential for the maintenance of immune homeostasis [5, 10, 11, 12]. In the liver, we previously reported that hepatocyte NIK regulates liver metabolism, injury, and regeneration [13, 14, 15, 16, 17]. Moreover, biliary NIK is also involved in liver disease progression, particularly cholestatic liver disease [4]. However, NIK downstream pathways, which mediate these functions in the liver, remain elusive. The role of biliary NIK in liver repair and liver disease resolution is unknown.

To further assess the pathological role of biliary NIK and its downstream pathways, we have generated cholangiocyte‐specific *NIK* transgenic mice (*NIK* ^*Tg*^) using a tamoxifen‐inducible CK19‐CreERT/loxp system. To model cholangiocyte reactivation and ductular reaction observed in human diseases, we treat mice with thioacetamide (TAA) or 3,5‐diethoxycarbonyl‐1,4‐dihydrocollidine (DDC) to induce biliary injury and regeneration, widely used rodent models of chronic liver disease. In this study, we report that cholangiocyte‐specific overexpression of NIK alone does not induce baseline liver injury. However, *NIK* ^*Tg*^ mice develop pronounced ductular reaction, liver inflammation, and liver fibrosis after exposure to DDC or TAA, leading to death. Remarkably, *NIK* ^*Tg*^ mice are resistant to liver injury resolution and fibrosis regression after cessation of liver toxin exposure. In cholangiocyte culture, genetic ablation of NIK reduces proliferation rates while increasing cell death, and reconstitution with recombinant NIK rescues the altered responses of *NIK*‐null cholangiocytes. Unexpectedly, reconstitution with NIK^G885R^, which is unable to activate the noncanonical IKKα/NF‐κB2 pathway [18, 19], also rescues the phenotype of *NIK*‐null cholangiocytes, albeit to a lesser extent. We have prepared conditioned medium from cholangiocytes, containing cholangiocyte‐secreted mediators—hereafter referred to as cholangiokines. We show that the cholangiokines activate both macrophage and hepatic stellate cells (HSCs) cultures. Activated HSCs are responsible for liver fibrosis [20, 21]. Deletion of *NIK* profoundly impairs cholangiokine secretion; importantly, reconstitution with either NIK or NIK^G885R^ rescues the defects although NIK^G885R^ exhibits reduced capability. Collectively, our data unveil a previously recognized pathological role of biliary NIK in shaping the susceptibility of the biliary system to liver stress and liver toxic substances. Furthermore, aberrant activation of biliary NIK hinders liver injury resolution and fibrosis regression, thereby contributing to poor prognosis.

## 2 Materials and Methods

### 2.1 Animals

*Rosa26‐loxp‐STOP‐loxp‐NIK* and *CK19‐CreERT* mice were described previously [4, 22]. *NIK* ^*Tg*^ mice (C57BL/6J background) were generated by crossing *Rosa26‐loxp‐STOP‐loxp‐NIK* with *CK19‐CreERT* mice. *Rosa26‐loxp‐STOP‐loxp‐NIK* ^*Tg/Tg*^;*CK19‐CreERT* ^*+/−*^ mice (8–9 weeks) were treated with tamoxifen (100 mg/kg body weight, once daily for 3 consecutive days, i.p.) to obtain *NIK* ^*Tg*^ mice. A corn oil vehicle was used as control (*NIK* ^*Con*^). Mice were housed on a 12‐h light–dark cycle and at 25°C ambient temperature in the Unit for Laboratory Animal Medicine at the University of Michigan (ULAM) and fed *ad libitum* a chow diet (9% fat in calories; TestDiet, St. Louis, MO).

### 2.2 Ethics Statement

Animal experiments were conducted following the protocols approved by the University of Michigan Institutional Animal Care and Use Committee (IACUC) and complied with all relevant ethical regulations.

### 2.3 TAA and DDC Treatments

Two weeks after tamoxifen treatment, *NIK* ^*Tg*^ and *NIK* ^*Con*^ male mice (10–11 weeks) were treated for 6 weeks with TAA (500 mg/L in drinking water; TAA: Sigma‐Aldrich Cat# 163678) or DDC (0.5% chow diet powder—LabDiet 5001; DDC: Sigma‐Aldrich Cat# 137030). Mice were euthanized after 4 weeks of TAA or DDC treatment, and livers and other tissues were harvested for in vitro studies. In a separate cohort, *NIK* ^*Tg*^ and *NIK* ^*Con*^ male mice were treated with TAA for 4 weeks, followed by a 2‐week recovery without TAA administration. For mortality experiments, mice were treated with TAA or DDC for 6 weeks.

### 2.4 Plasma ALT and ALP Assay

Blood samples were collected from tail veins. Plasma ALT and ALP levels were measured using commercial kits (Pointe Scientific Inc., Canton, MI; ALT: A7526625; ALP: A7516150).

### 2.5 Cell Cultures and Transfection

H69, HuCC‐T1, and LX2 cells were cultured in a 5% CO_2_ incubator at 37°C in Dulbecco's modified Eagle's medium (DMEM) (Gibco) supplemented with 10% (v/v) fetal bovine serum (FBS) (Sigma‐Aldrich), 100 U/mL penicillin, and 100 μg/mL streptomycin (Gibco, 15 140 122). Medium was changed every other day. HuCC‐T1^NIK−/−^ cells (80%–90% confluence) were transfected with NIK, NIK^ΔC287^, or NIK^G885R^ expression vectors using Lipofectamine 3000 liposome reagents (Lipofectamine 3000 Transfection Reagent, Invitrogen, Cat# L3000008). After 12 h, transfection medium was removed and replaced with fresh culture medium, and experiments were performed 24 h later.

### 2.6 CRISPR/Cas9‐Based *NIK* Knockout

The *NIK/MAP3K14* gene was deleted in the HuCC‐T1 cells using the CRISPR/Cas9 gene‐editing plasmid lentiCRISPR v2‐Blast (Addgene #83480) and guide RNAs (gRNAs) designed for *MAP3K14*. Using an online tool (https://portals.broadinstitute.org/gppx/crispick/public), we designed 2 gRNAs: 5′‐CACCGTGGAATACCTCCACTCACGA‐3′ and 3′‐AAACTCGTGAGTGGAGGTATTCCAC‐5′. The gRNAs were inserted into lentiCRISPR v2‐Blast plasmids at the BsmBI site. To generate lentiviral particles, 293FT cells were transfected with the lentiCRISPR v2‐Blast gRNA vectors (10 μg), psPAX2 (5 μg) (Plasmid #12260), and pMD2.G (5 μg) (Addgene #12259) and grown in DMEM supplemented with 10% FBS. After 12 h of culture, the transfection medium was replaced with fresh DMEM and collected 48 h later. The virus‐containing medium was then filtered through a 0.44 μm filter and stored at −80°C. HuCC‐T1 cells were grown in a medium composed of 1 mL viral medium and 1 mL normal growth medium, supplemented with 10 μg/mL polybrene. After 8 h of transduction, the medium was replaced with fresh culture medium, and the cells were incubated for an additional 48 h. Transduced cells were then selected using blasticidin (10 μg/mL) for 3 days. Individual clones were isolated by serial dilutions. To assess gene editing efficiency and identify HuCC‐T1^NIK−/−^ clones, a T7 Endonuclease I (T7E1) assay was performed to detect cleavage of the targeted *MAP3K14* gene.

### 2.7 Conditioned Medium (CM) Preparation

NIK inhibitor Compound 33 (C33) was synthesized and purified by Shanghai Institute of Materia Medica (Chinese Academy of Sciences, Shanghai) and suspended in a vehicle containing 10% PEG400 (polyethylene glycol, molecular weight 400, Sigma‐Aldrich P3265) and 3% Cremophor EL (Sigma‐Aldrich, C5135) as described previously [4]. Confluent H69 cells were treated with 2 μM C33 for 24 h, washed with PBS, and then cultured in fresh DMEM for an additional 12 h. Supernatant—CM—was collected, and dead cells were removed from CM by centrifugation. Similarly, confluent HuCC‐T1 or its derived cells were washed with PBS and grown in FBS‐free DMEM. CMs were collected 12 h later and purified through centrifugations.

### 2.8 BMDM Isolation and Culture

Bone marrow cells were isolated from the femur and tibia of 6–7 weeks male C57BL/6J mice and differentiated to mature macrophages for 7 days as described previously [23]. Briefly, cells were maintained in DMEM with 10% FBS, containing 10 ng/mL M‐CSF (Perprotech, 315–02). On day 7, conditioned medium collected from H69 or HuCC‐T1 cells was added for CM treatment for 12 h.

### 2.9 Cholangiokine Bioassays

LX2 or BMDM cells were washed with PBS and cultured in a mixture of CM and fresh DMEM at a 1:1 ratio. After 12 h, cells were harvested to prepare total RNA and lysates for qPCR and immunoblotting assays, respectively.

### 2.10 Western Blot

Liver tissue samples were homogenized or cell lysates were lysed in 2% sodium dodecyl sulfate (SDS) buffer containing protease inhibitor cocktail (Roche #04693116001) and phosphatase inhibitor cocktail (Roche #04693132001), using TissueLyser II (QIAGEN, Valencia, CA). Cell cultures were lysed in 2% SDS lysis buffer supplemented with protease inhibitor and phosphatase inhibitor cocktails. Proteins (10–30 μg) were separated by SDS‐PAGE and then transferred onto a nitrocellulose transfer membrane (NC, 0.2 μm) using the wet transfer method. Membranes were blocked with 5% fat‐free milk in TBST (Tris‐buffered saline plus 0.1% Tween‐20) for one hour at ambient temperature, and the membranes were incubated in primary antibodies overnight at 4°C. The next day, the membranes were washed in TBST (3 × 10 min) and then incubated with HRP‐conjugated secondary antibodies for one hour at ambient temperature. After TBST washes (3 × 10 min), Pierce ECL western blotting substrate was added onto the membrane and incubated for two minutes to develop the chemiluminescent signal. Antibodies were listed in Table S1.

### 2.11 Histology

Freshly isolated liver tissue was fixed in 4% paraformaldehyde for 24 h at ambient temperature. Tissues were embedded in paraffin, sectioned at 8 μm thickness, deparaffinized, and rehydrated through graded concentrations of ethanol in water. Liver paraffin sections were stained with 0.1% Sirius‐red (Sigma‐Aldrich, 365 548) and 0.1% Fast‐green (Sigma‐Aldrich, F7252) in saturated picric acid. Liver frozen sections were prepared using a Leica cryostat (Leica Biosystems Nussloch GmbH, Nussloch, Germany), and then fixed in 4% paraformaldehyde for 10 min, blocked with 5% normal goat serum (Gibco) supplemented with 1% BSA for 1 h at ambient temperature. Then the liver sections were incubated with the indicated antibodies overnight at 4°C. Liver sections or cell cultures were stained with TUNEL reagents using an In Situ Cell Death Detection Kit (Roche Diagnostics, Indianapolis, IN, 11684817910), following the manufacturer's instructions. Liver paraffin sections were stained with 0.1% Sirius‐red (Sigma, 365548) and 0.1% Fast‐green (Sigma, F7252) (in saturated picric acid) as we reported previously [24]. Antibodies were listed in Table S1. Sirius Red^+^ and α‐SMA^+^ areas (10× magnification) were quantified using ImageJ in a blinded manner. For each liver sample, five non‐overlapping fields of view were randomly selected for quantification, avoiding large vessels or tissue edges. This strategy minimized potential bias arising from uneven tissue sectioning.

### 2.12 RNA Extraction and qPCR

Total RNA was extracted from liver tissues or cell cultures using TRIzol (Invitrogen), and used to prepare cDNA using ABScript II cDNA First‐Strand Synthesis Kit (ABclonal Technology). Gene expression was assessed by qPCR using 2X Universal SYBR green fast qPCR mix (ABclonal Technology, Cat# RK21203) and Mastercycler qPCR system (Eppendorf). Subsequently, relative mRNA levels were calculated using the comparative CT method (ΔΔCT) and normalized to *18S rRNA* or Glyceraldehyde‐3‐phosphate dehydrogenase (GAPDH) mRNA levels. The average values of the control group were set as one, and all the results were presented as the relative mRNA expression. Primers were listed in Table S2.

### 2.13 Statistical Analysis

All data (collected from animals or cell culture) were randomized. Data were analyzed using GraphPad Prism v10.4.1 and presented as mean values ± SD. Difference was analyzed by a two‐tailed Student's *t*‐test for two groups or two‐way ANOVA/Bonferroni posttest for multiple groups. A *p* value less than 0.05 was considered statistically different.

## 3 Results

### 3.1 Cholangiocyte‐Specific Overexpression of NIK Exacerbates Toxicant‐Induced Liver Injury

**FIGURE 1:**
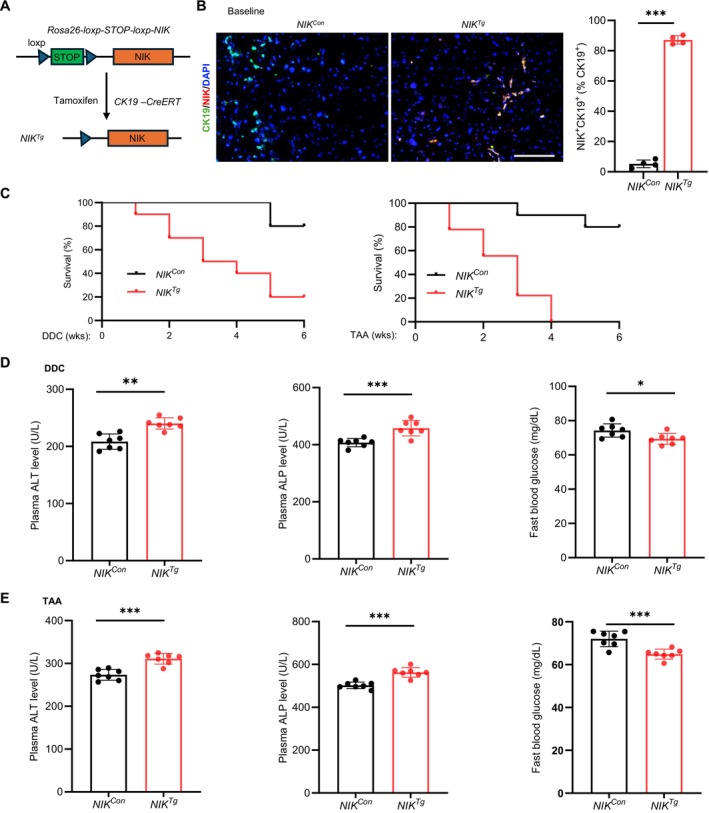
Cholangiocyte‐specific overexpression of NIK exacerbates toxicant‐induced fatal liver injury. (A) Generation of *NIK* ^*Tg*^ mice. (B) Liver sections were prepared at 16 weeks of age and stained with antibodies against CK19 and NIK. CK19^+^NIK^+^ cells were normalized to total CK19^+^ cholangiocytes (*n* = 4 mice per group). Scale bar: 100 μm. (C) Survival rates following DDC or TAA treatment (*n* = 10 mice per group). (D) Plasma ALT, ALP and overnight fasting blood glucose levels 4 weeks after DDC treatment (*n* = 7 mice per group). (E) Plasma ALT, ALP and overnight fasting blood glucose levels 4 weeks after TAA treatment (*n* = 7 mice per group). Data are presented as mean ± SD. *p < 0.05, **p < 0.01, ***p < 0.001 by a two‐tailed Student's *t*‐test.

To study the pathological role of biliary NIK in vivo, we generated cholangiocyte‐specific *NIK* transgenic mice (*NIK* ^*Tg*^). We previously characterized a Cre‐inducible *Rosa26‐loxp‐STOP‐loxp‐NIK* strain containing a *loxp‐STOP‐loxp‐NIK* transgene in the *Rosa26* allele [16, 22]. Cre recombinase is expected to excise the *STOP* sequence, thus activating the *NIK* transgene. *Rosa26‐loxp‐STOP‐loxp‐NIK* mice were crossed with tamoxifen‐inducible, cholangiocyte‐specific *CK19‐CreERT* drivers. The progenies (*loxp‐STOP‐loxp‐NIK* ^*Tg/Tg*^;*CreERT* ^*+/−*^) were treated with tamoxifen (activating CreERT) to activate the *NIK* transgene specifically in cholangiocytes, generating *NIK* ^*Tg*^ mice (Figure 1A). A corn oil vehicle was used as control (*NIK* ^*Con*^). As expected, endogenous NIK was below the detectable level in *NIK* ^*Con*^ mice, whereas in *NIK* ^*Tg*^ mice, NIK was readily detected and colocalized with the cholangiocyte marker cytokeratin 19 (CK19) (Figure 1B). *NIK* ^*Tg*^ mice were grossly normal under normal conditions, suggesting that the overexpression of biliary NIK alone is insufficient to induce liver injury.

To induce biliary injury, *NIK* ^*Tg*^ and *NIK* ^*Con*^ littermates were treated for 6 weeks with either TAA (500 mg/L in drinking water) or DDC (0.5% in diet). Both toxicants progressively caused death; however, mortality was substantially higher in *NIK* ^*Tg*^ than in *NIK* ^*con*^ mice (Figure 1C). Plasma alanine aminotransferase (ALT) and alkaline phosphatase (ALP) levels, liver injury markers, were significantly elevated in *NIK* ^*Tg*^ mice within 4 weeks of DDC or TAA treatment (Figure 1D,E). Fasting blood glucose levels, a liver function index [25], were significantly reduced in *NIK* ^*Tg*^ mice following DDC or TAA treatment (Figure 1D,E). These results indicate that aberrant biliary NIK expression profoundly increases hepatic susceptibility to toxic insults, ultimately leading to mortality.

### 3.2 NIK Cell‐Autonomously Promotes Cholangiocyte Growth, Survival, and Ductular Reaction

**FIGURE 2:**
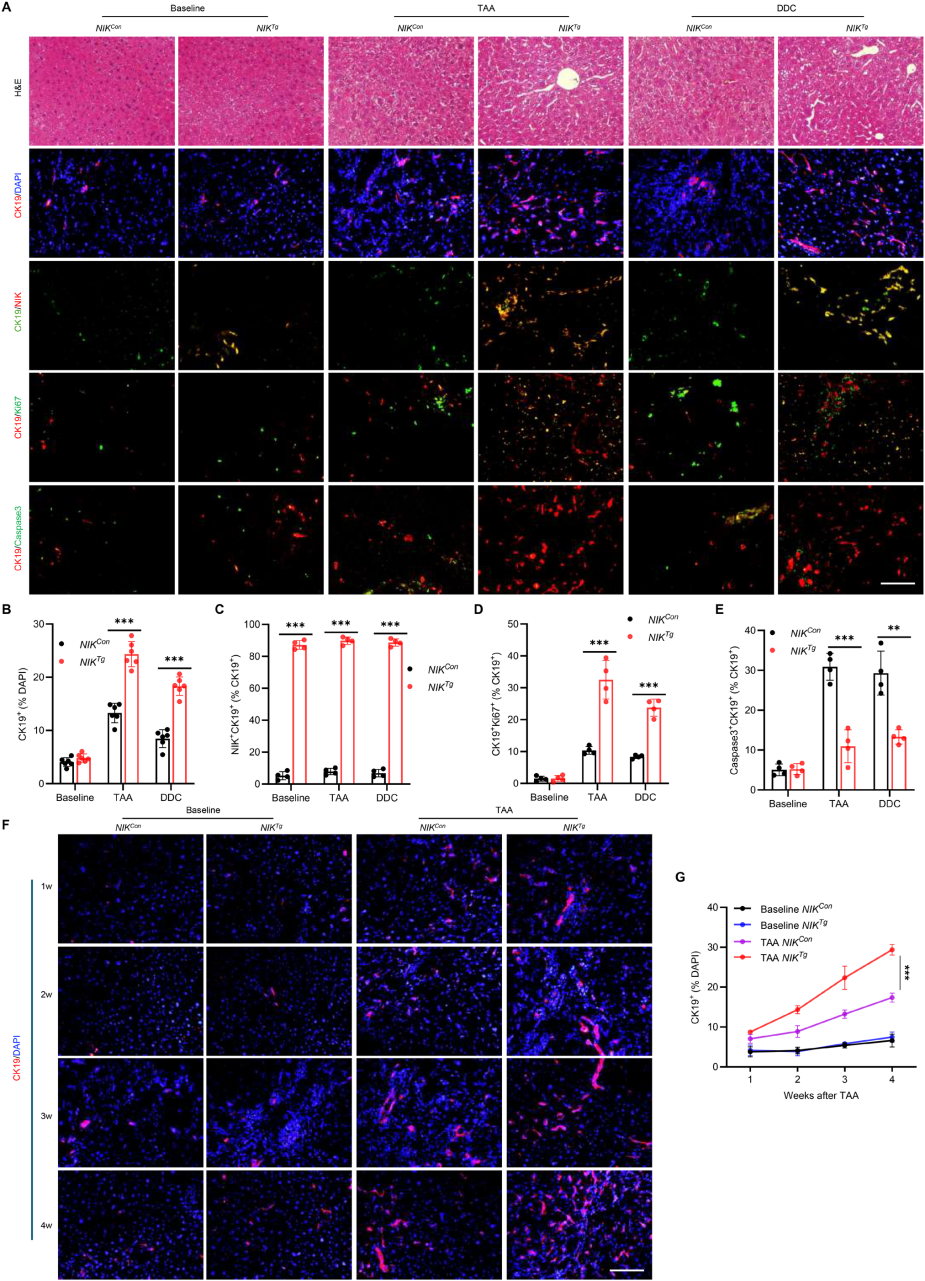
Biliary NIK promotes cholangiocyte proliferation, survival, and ductular reaction. (A–E) Male mice were treated with TAA or DDC for 4 weeks, and liver sections were stained with the indicated reagents. (A) Representative images. Scale bar: 100 μm. (B–E) (B) CK19^+^ cholangiocytes (*n* = 6 mice per group, normalized to total DAPI^+^ cells), (C) NIK^+^CK19^+^ cells (*n* = 4 per group, normalized to total CK19^+^ cholangiocytes), (D) Ki67^+^CK19^+^ proliferating cholangiocytes (*n* = 4 per group, normalized to total cholangiocytes), and (E) Caspase3^+^CK19^+^ apoptotic cholangiocytes (*n* = 4 per group, normalized to total cholangiocytes) were quantified. (F, G) Male mice were treated with TAA for 1–4 weeks, and liver sections were stained with anti‐CK19 antibody. (F) Representative images. Scale bar: 100 μm. (G) CK19^+^ cholangiocytes were quantified and normalized to total cells (*n* = 4 mice per group). Data are presented as mean ± SD. **p < 0.01, ***p < 0.001 by a two‐tailed Student's *t*‐test (B–E) and a two‐way ANOVA (G).

We set out to investigate the underlying mechanisms of NIK‐caused liver injury. The baseline cholangiocyte number, assessed by anti‐CK19 antibody, was comparable between *NIK* ^*Con*^ and *NIK* ^*Tg*^ mice; however, CK19^+^ cholangiocytes were profoundly increased in *NIK* ^*Tg*^ mice after TAA or DDC treatment (Figure 2A,B). Notably, newly generated cholangiocytes expressed NIK (CK19^+^NIK^+^) (Figure 2A,C), suggesting that they arose from NIK‐expressing cholangiocytes. These findings indicate that NIK upregulation increases the biliary susceptibility to insults, resulting in a pathogenic ductular reaction.

To delineate how NIK promotes ductular reaction, we assessed cholangiocyte proliferation and death in *NIK* ^*Tg*^ mice. NIK overexpression significantly increased cholangiocyte proliferation (CK19^+^Ki67^+^) in *NIK* ^*Tg*^ mice compared to *NIK* ^*Con*^ littermates following DDC or TAA treatment (Figure 2A–E). Moreover, NIK also suppressed cholangiocyte death in *NIK* ^*Tg*^ mice, as evidenced by a decreased number of CK19^+^Caspase 3^+^ cells (Figure 2A–E). To examine the time course of ductular reaction, we counted cholangiocytes weekly for 4 weeks after TAA treatment. Baseline cholangiocyte levels were low and comparable between *NIK* ^*Con*^ and *NIK* ^*Tg*^ mice. TAA treatment progressively increased cholangiocyte number in both groups, but to a significantly higher degree in *NIK* ^*Tg*^ mice (Figure 2F,G). Collectively, these results suggest that NIK pathways promote ductular reaction by both enhancing cholangiocyte proliferation and suppressing its death.

### 3.3 Biliary NIK Promotes Liver Inflammation

**FIGURE 3:**
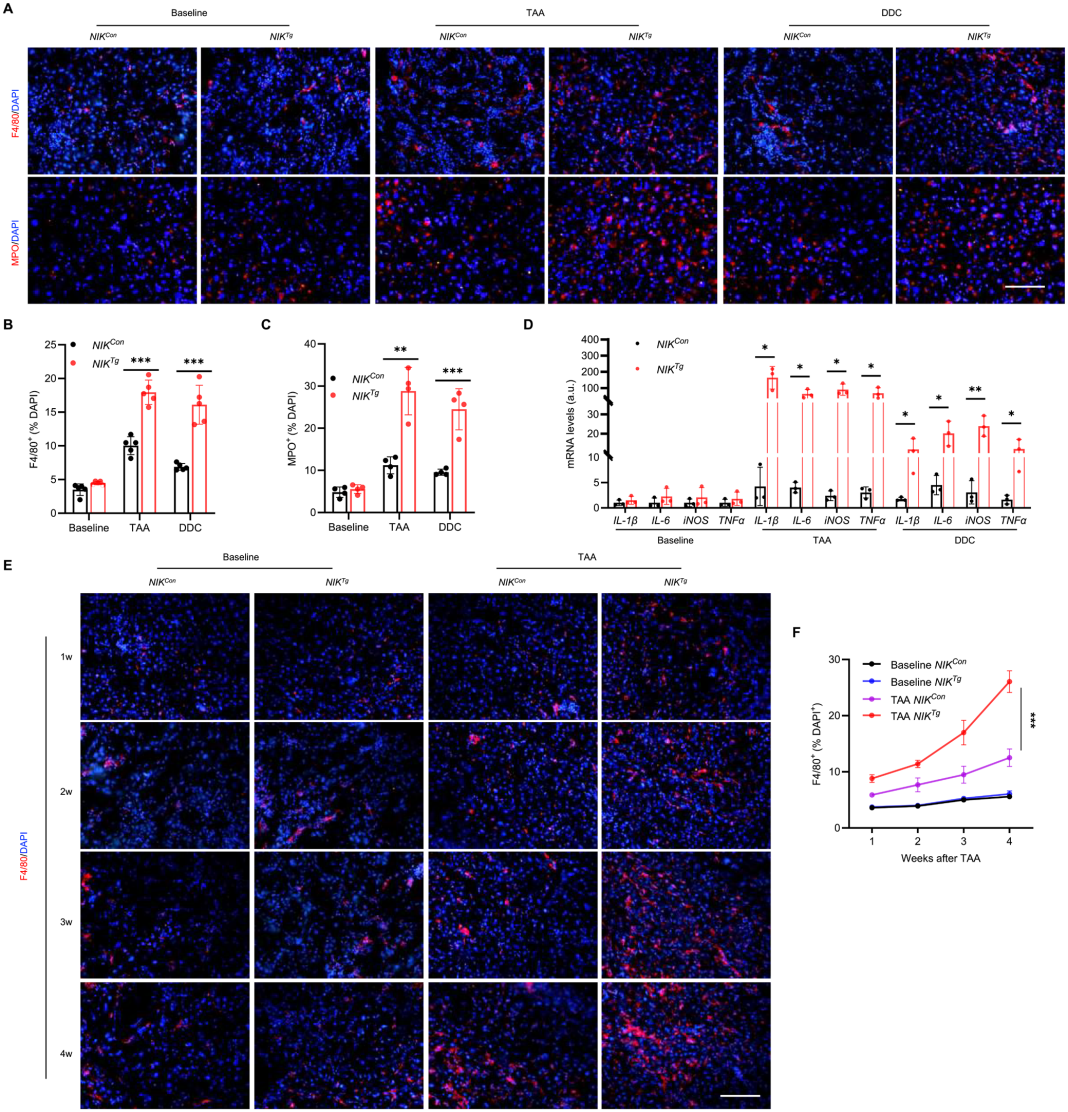
Biliary NIK enhances toxicant‐induced liver inflammation. (A–D) Male mice were treated with TAA or DDC for 4 weeks. (A–C) Liver sections were stained with antibodies against F4/80 and MPO. F4/80^+^ macrophages/Kupffer cells (*n* = 5 mice per group) and MPO^+^ neutrophils (*n* = 4 per group) were quantified and normalized to total cells. Scale bar: 100 μm. (D) Liver gene expression was measured by qPCR (normalized to *18S rRNA* expression, *n* = 3 mice per group). a.u., arbitrary unit. (E, F) Male mice were treated with TAA for 4 weeks and liver sections were stained with anti‐F4/80 antibody. F4/80^+^ macrophages/Kupffer cells were quantified and normalized to total cells (*n* = 4 mice per group). Scale bar: 100 μm. Data are presented as mean ± SD. Data in (B–D) were analyzed using a two‐tailed Student's *t*‐test for comparisons. *p < 0.05, **p < 0.01, ***p < 0.001 by a two‐tailed Student's *t*‐test (B–D) and a two‐way ANOVA (F).

We next sought to examine the impact of NIK‐accelerated ductular reaction on liver inflammation. *NIK* ^*Tg*^ and *NIK* ^*Con*^ littermates were treated with DDC or TAA for 4 weeks, and F4/80^+^ macrophage/Kupffer cells and myeloperoxidase (MPO)^+^ neutrophils were histologically analyzed in liver sections. Although their baseline numbers were comparable between the two groups, TAA or DDC treatment increased both F4/80^+^ and MPO^+^ cells to a significantly higher level in *NIK* ^*Tg*^ mice relative to *NIK* ^*Con*^ mice (Figure 3A–C). To extend these findings, we measured liver expression of proinflammatory mediators by qPCR. TNFα, IL‐1β, IL‐6, and inducible nitric oxide synthase (iNOS) expressions were significantly higher in TAA‐ or DDC‐treated *NIK* ^*Tg*^ mice relative to *NIK* ^*Con*^ littermates (Figure 3D). Time course analysis revealed that TAA progressively increased F4/80^+^ macrophages/Kupffer cells in both *NIK* ^*Con*^ and *NIK* ^*Tg*^ mice, but the increase was significantly greater in *NIK* ^*Tg*^ mice (Figure 3E,F).

### 3.4 Biliary NIK Promotes HSC Activation and Liver Fibrosis

**FIGURE 4:**
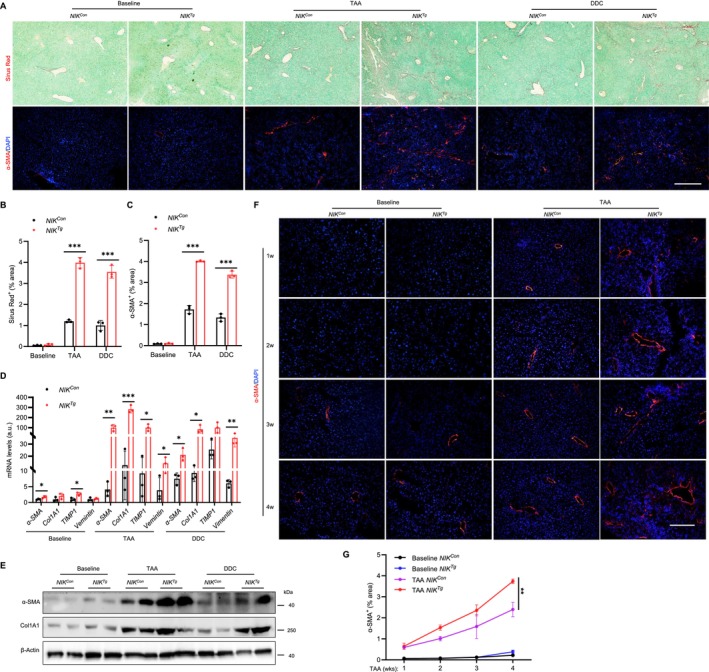
Biliary NIK enhances toxicant‐induced liver fibrosis. (A–G) Male mice were treated with TAA or DDC for 4 weeks. Liver sections were stained with the indicated reagents. (A) Representative images. Sirius Red staining (upper panel) and immunofluorescence staining using antibodies against α‐SMA (lower panel) in liver sections. Scale bar: 400 μm. (B, C) Sirius Red (*n* = 3 mice per group, normalized to the total area) and α‐SMA^+^ HSC areas (*n* = 3 per group, normalized to the total area) were quantified. (D) Liver mRNA expression was measured by qPCR and normalized to *18S rRNA* levels (*n* = 3 mice per group). a.u., arbitrary unit. (E) Western blot analysis for the liver tissue. (F, G) Male mice were treated with TAA for 4 weeks and liver sections were stained with α‐SMA antibody. α‐SMA^+^ HSC areas were quantified and normalized to the total area (*n* = 4 mice per group). Scale bar: 200 μm. Data are presented as mean ± SD. Data in (B–D) were analyzed using a two‐tailed Student's *t*‐test and a two‐way ANOVA (G) for comparisons. *p < 0.05, **p < 0.01, ***p < 0.001.

Ductular reaction and inflammation are often associated with liver fibrosis [21], prompting us to investigate HSC activation and fibrosis progression in *NIK* ^*Tg*^ mice. Mice were treated with TAA or DDC for 4 weeks, and HSC activation was defined by α‐smooth muscle actin (α‐SMA) and collagen type I alpha 1 chain (Col1a1) expressions. Liver fibrosis was measured by Sirius red staining of liver sections. Baseline liver fibrosis was undetectable in both *NIK* ^*Tg*^ and *NIK* ^*Con*^ mice. TAA or DDC stimulated HSC activation (α‐SMA^+^ cells) and liver fibrosis (Sirius Red^+^ areas) in both groups, but to a greater extent in *NIK* ^*Tg*^ mice (Figure 4A–C). Fibrosis was detected mainly in portal and periportal areas. Consistently, liver expression of pro‐fibrotic genes—including *α‐SMA*, *Col1a1*, and tissue inhibitor of metalloproteinases 1 (TIMP1)—was significantly elevated in *NIK* ^*Tg*^ mice compared to *NIK* ^*Con*^ mice following treatment with either TAA or DDC (Figure 4D). Liver α‐SMA and Col1a1 protein levels were also substantially higher in *NIK* ^*Tg*^ than in *NIK* ^*Con*^ mice (Figures 4E and S1). Time course analysis revealed that TAA progressively increased HSC activation in both *NIK* ^*Con*^ and *NIK* ^*Tg*^ mice, and the increase was significantly more pronounced in *NIK* ^*Tg*^ mice (Figure 4F,G).

### 3.5 Biliary NIK Enhances Secretion of Pro‐Inflammatory and Pro‐Fibrotic Cholangiokines

**FIGURE 5:**
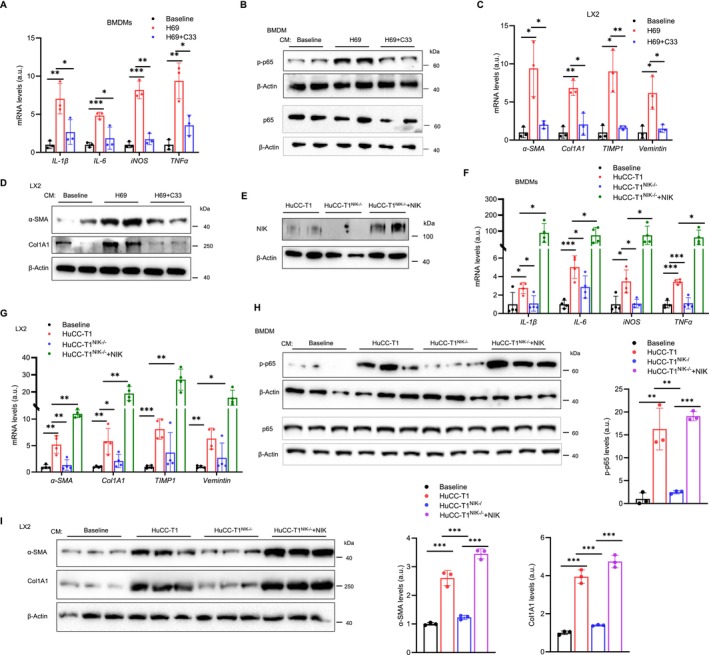
Biliary NIK promotes cholangiokine secretion. (A, B) BMDMs were treated with the indicated CMs for 24 h (A) Cytokine expression was measured by qPCR and normalized to *18S rRNA* expression (*n* = 3 repeats per group). a.u., arbitrary unit. (B) BMDM extracts were immunoblotted with the indicated antibodies. (C, D) LX2 cells were treated with the indicated CMs for 24 h. (C) LX2 gene expression was assessed by qPCR and normalized to *GAPDH* expression (*n* = 3 repeats per group). (D) LX2 extracts were immunoblotted with the indicated antibodies. (E) NIK was deleted in HuCC‐T1 cells using CRISPR/Cas9. HuCC‐T1^NIK−/−^ cells were then transfected with NIK plasmids to restore NIK. Cell extracts were immunoblotted with anti‐NIK antibody to validate NIK expression. (F–I) BMDMs and LX2 cells were treated with the indicated CMs for 24 h. (F, G) Gene expression was measured by qPCR and normalized to *18S rRNA* levels for BMDMs and to *GAPDH* levels for LX2 (*n* = 4 repeats per group). (H, I) Cells extracts were immunoblotted with the indicated antibodies. p65 phosphorylation was quantified and normalized to total p65 levels. α‐SMA and Col1A1 levels were normalized to β‐actin levels. *n* = 3 per group. Data are presented as mean ± SD. *p < 0.05, **p < 0.01, ***p < 0.001 by a two‐tailed Student's *t*‐test.

We hypothesized that NIK enhances the ability of cholangiocytes to secrete mediators (referred to as cholangiokines) that promote liver inflammation and fibrosis. To test this idea, H69 cells, a human cholangiocyte line, were treated with NIK inhibitor Compound 33 (C33) or an empty vehicle (control). C33 potently inhibits NIK in vitro and in vivo [4]. Conditioned medium (CM), which contains cholangiokines, was collected from C33‐treated (CM‐C33) and control cells (CM‐Con). Bone marrow‐derived macrophages (BMDMs) were stimulated with CM‐C33 or CM‐Con, and their expression of cytokines was measured by qPCR. CM‐Con, but not CM‐C33, markedly upregulated *IL‐1β, IL‐6, iNOS*, and *TNFα* expressions (Figure 5A). CM‐Con but not CM‐C33 also stimulated phosphorylation of p65, a BMDM activation marker (Figures 5B and S2A). To examine the role of cholangiokines in HSC activation, LX2 cells, a human HSC line, were treated with CMs. CM‐Con, but not CM‐C33, significantly increased the expression of *α‐SMA*, *Col1a1*, *TIMP1*, and *Vimentin* (Figure 5C). Consistently, α‐SMA and Col1a1 protein levels were also increased by CM‐Con but not CM‐C33 (Figures 5D and S2B).

To further validate the role of NIK in cholangiokine secretion, the *NIK* gene was deleted in HuCC‐T1 cells (HuCC‐T1^NIK−/−^)―a human intrahepatic cholangiocarcinoma line—using CRISPR/Cas9. We generated two independent HuCC‐T1^NIK−/−^ lines (Figure S3A). To examine NIK pathways, these cells were treated with cytokine TWEAK. TWEAK increased NF‐κB2 p52 levels in HuCC‐T1 but not HuCC‐T1^NIK−/−^ cells (Figure S3B). NIK protein was detected in HuCC‐T1 but not HuCC‐T1^NIK−/−^ cells (Figure 5E). Recombinant NIK was reintroduced into HuCC‐T1^NIK−/−^ cells using transfection (Figures 5E and S3C). We prepared CMs from these human cholangiocytes and assessed their cholangiokine activities using BMDMs (inflammation) and LX2 cells (fibrosis). CM from HuCC‐T cells, but not CM from HuCC‐T1^NIK−/−^ cells, significantly upregulated cytokine expression in BMDMs (e.g., *IL‐1β, iNOS*, and *TNFα*) and fibrotic gene expression in LX2 cells (e.g., *α‐SMA*, *Col1a1*, *TIMP1* and *Vimentin*), respectively (Figure 5F,G). NIK reconstitution fully rescued the ability of CM from NIK‐transfected HuCC‐T1^NIK−/−^ cells to activate BMDMs and LX2 cells (Figure 5F,G). Notably, CM from NIK‐reconstituted HuCC‐T1^NIK−/−^ cells demonstrated an increased capability to activate BMDMs and LX2 cells compared to CM from HuCC‐T1 cells, likely due to increased NIK levels in these cells (Figure 5F,G). Consistently, CMs from HuCC‐T1 cells, or CM from NIK‐transfected HuCC‐T1^NIK−/−^ cells, increased p65 phosphorylation in BMDMs (Figure 5H) and enhanced expression of α‐SMA and Col1a1 in LX2 cells (Figure 5I) compared with CM from HuCC‐T1^NIK−/−^ cells. These results suggest that biliary NIK critically regulates the crosstalk of cholangiocytes with immune cells and HSCs through promoting cholangiokine secretion.

### 3.6 The NIK/IKKα/NF‐κB2 Pathway Promotes Cholangiocyte Proliferation and Survival

**FIGURE 6:**
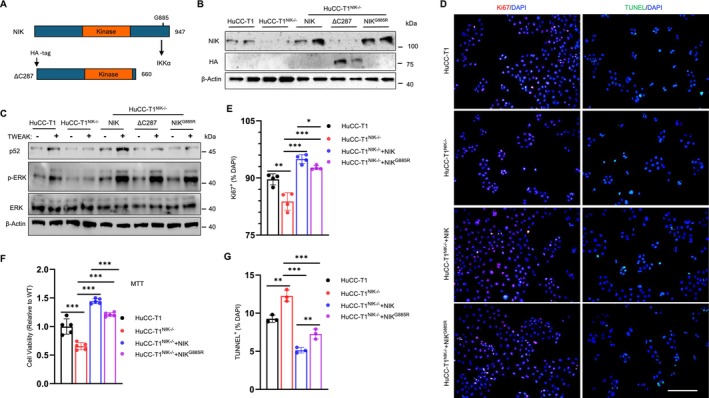
NIK promotes cholangiocyte growth by both IKKα‐dependent and ‐independent mechanisms. (A) Schematic representation of NIK and HA‐tagged NIK^ΔC287^. (B) HuCC‐T1^NIK−/−^ cells were transfected with NIK, HA‐NIK^Δ287^, or NIK^G885R^ plasmids for 48 h. Cell extracts were immunoblotted with anti‐NIK and anti‐HA antibodies. (C) HuCC‐T1, HuCC‐T1^NIK−/−^, and HuCC‐T1^NIK−/−^ cells with reconstitution of NIK, HA‐NIK^Δ287^, or NIK^G885R^ were starved overnight and stimulated with 10 ng/mL TWEAK for 60 min. Cell extracts were immunoblotted with the indicated antibodies. p52 and β‐actin were from the same blot, whereas pERK and ERK were obtained from duplicate blots. (D, E) HuCC‐T1 and its derived cells were stained with anti‐Ki67 antibody (assessing proliferation) and TUNEL reagent (cell death). Ki67^+^ cell numbers were quantified and normalized to total cell numbers (*n* = 4 repeats per group). Scale bar: 200 μm. (F) Cell viability in MTT assays (*n* = 5 per group). (G) TUNEL^+^ cell numbers in D were normalized to total cell numbers (*n* = 4 per group). Data are presented as mean ± SD. *p < 0.05, **p < 0.01, ***p < 0.001 by a two‐tailed Student's *t*‐test.

To test if IKKα acts downstream of NIK to enhance cholangiocyte expansion, we generated NIK^ΔC287^ and NIK^G885R^ mutants—defective in binding to IKKα (Figure 6A) [22]. HuCC‐T1^NIK−/−^ cells were transfected with plasmids expressing NIK, HA‐tagged NIK^ΔC287^, or NIK^G885R^. We confirmed expression of recombinant NIK, NIK^ΔC287^ and NIK^G885R^―modestly above endogenous NIK levels (Figure 6B). TWEAK stimulation increased p52 levels (activation of the NIK/IKKα pathway) in both HuCC‐T1 cells and NIK‐transfected HuCC‐T1^NIK−/−^ cells, but not in HuCC‐T1^NIK−/−^ cells reconstituted with either NIK^ΔC287^ or NIK^G885R^ (Figure 6C). Unexpectedly, TWEAK stimulated phosphorylation of ERK1/2 in HuCC‐T1^NIK−/−^ cells reconstituted with NIK, NIK^ΔC287^, or NIK^G885R^ (Figure 6C). These results demonstrate that neither NIK^ΔC287^ nor NIK^G885R^ is able to activate the IKKα/NF‐κB2 pathway.

Deletion of *NIK* significantly suppressed human cholangiocyte proliferation and survival, as evidenced by a reduced number and viability of Ki67^+^ HuCC‐T1^NIK−/−^ cells compared to HuCC‐T1 cells (Figure 6D–F). Reintroduction of NIK fully rescued the defects in HuCC‐T1^NIK−/−^ cells, and NIK^G885R^ partially restored HuCC‐T1^NIK−/−^ cell proliferation and survival (Figure 6D–F). *NIK* deletion enhanced cell death, as indicated by a significantly higher number of TUNEL^+^ cells in HuCC‐T1^NIK−/−^ cells compared to HuCC‐T1 controls (Figure 6D,G). NIK reconstitution dramatically suppressed cell death in HuCC‐T1^NIK−/−^ cells, reducing TUNEL^+^ cell counts to levels below those of HuCC‐T1 controls. Notably, NIK^G885R^ exhibited a reduced capability, compared to NIK, to rescue cell death in HuCC‐T1^NIK−/−^ cells (Figure 6G). These results suggest that NIK enhances cholangiocyte proliferation and survival by both IKKα‐dependent and ‐independent mechanisms.

### 3.7 The NIK/IKKα/NF‐κB2 Pathway Promotes Cholangiokine Secretion

**FIGURE 7:**
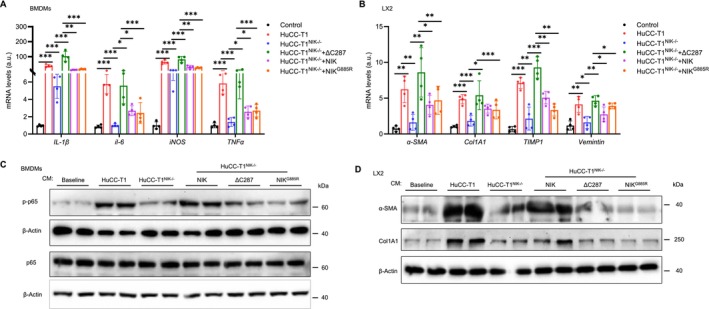
NIK promotes cholangiokine secretion by both IKKα‐dependent and ‐independent mechanisms. BMDMs and LX2 cells were treated with the indicated CMs for 24 h. Gene expression was measured by qPCR and normalized to *18S rRNA* levels for BMDMs and *GAPDH* levels for LX2 (*n* = 4 repeats per group). Cell extracts were immunoblotted with the indicated antibodies. (A, B) Gene expression. a.u., arbitrary unit. (C, D) Immunoblotting of cell extracts. Data are presented as mean ± SD. *p < 0.05, **p < 0.01, ***p < 0.001 by a two‐tailed Student's *t*‐test.

To determine whether the NIK/IKKα/NF‐κB2 pathway regulates cholangiocyte secretion, we prepared CMs from HuCC‐T1 cells, HuCC‐T1^NIK−/−^ cells, and HuCC‐T1^NIK−/−^ cells reconstituted with NIK, NIK^ΔC287^, or NIK^G885R^. We assessed cholangiokines in these CMs using BMDMs and LX2 cells as reporters. NIK deficiency in HuCC‐T1^NIK−/−^ cells profoundly impaired their secretion of cholangiokines that stimulate *IL‐6* and *TNFα* expression in BMDMs, as well as *α‐SMA*, *Col1a1*, and *TIMP1* expression in LX2 cells (Figure 7A,B). Reconstitution with NIK in HuCC‐T1^NIK−/−^ cells fully rescued cholangiokine secretion, whereas NIK^ΔC287^ and NIK^G885R^ exhibited partial rescuing effects (Figure 7A,B). Consistently, CMs from NIK^ΔC287^‐ and NIK^G885R^‐reconstituted cells showed a reduced ability to stimulate p65 phosphorylation in BMDMs and to increase α‐SMA and Col1a1 levels in LX2 cells (Figures 7C,D and S3D). These results suggest that NIK enhances cholangiokine secretion by both IKKα‐dependent and ‐independent mechanisms.

### 3.8 Biliary NIK Halts Liver Injury Resolution and Fibrosis Regression

**FIGURE 8:**
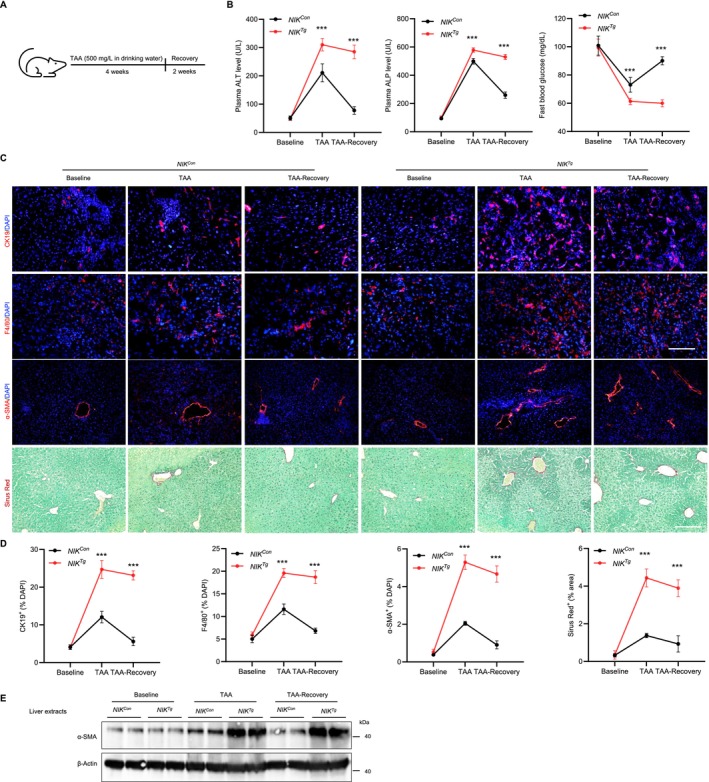
Biliary NIK hinders liver injury resolution and fibrosis regression. (A) Experimental regimen. (B) Plasma ALT, ALP and overnight fasting blood glucose levels (*n* = 6 mice per group). (C) Representative images of liver sections stained with the indicated reagents. Scale bar: 100 μm for H&E, CK19 and F4/80, 200 μm for α‐SMA and Sirus Red. (D) Quantifications from C. CK19^+^ cholangiocytes (normalized to DAPI^+^ cells): *N* = 4 per group, F4/80^+^ cells (normalized to DAPI^+^ cells): *N* = 3 per group, α‐SMA^+^ HSC area (normalized to the total area): *N* = 3 per group, and Sirius Red area (normalized to the total area): *N* = 3 per group. (E) Western blot analysis for the liver tissue. Data are presented as mean ± SD. ***p < 0.001 by a two‐tailed Student's *t*‐test (*NIK* ^*Tg*^ vs. *NIK* ^*Con*^).

We next investigated the role of biliary NIK in the resolution of liver injury. *NIK* ^*Con*^ and *NIK* ^*Tg*^ male mice were treated with TAA for 4 weeks, followed by a 2‐week recovery period (Figure 8A). TAA administration induced significant liver injury, as evidenced by marked elevations in plasma ALT and ALP levels (Figure 8B). Liver function was severely impaired, reflected by a dramatic reduction in overnight fasting blood glucose levels in TAA‐treated mice (Figure 8B). As expected, during the recovery phase, plasma ALT levels were returned to baseline in wild‐type *NIK* ^*Con*^ mice, and plasma ALP levels were also markedly reduced. Concomitantly, blood glucose levels rebounded, consistent with the resolution of TAA‐induced liver injury. In stark contrast, *NIK* ^*Tg*^ mice failed to recover. TAA‐induced elevations in plasma ALT and ALP, as well as pathogenic hypoglycemia, persisted in *NIK* ^*Tg*^ mice even after cessation of TAA treatment. Sustained hypoglycemia and liver dysfunction likely underlie the observed mortality in TAA‐ or DDC‐treated mice.

We next histologically examined liver injury in these mice. As expected, TAA administration in *NIK* ^*Con*^ mice promoted the expansion of CK19^+^ cholangiocytes, F4/80^+^ macrophages/Kupffer cells, and α‐SMA^+^ activated HSCs, and induced liver fibrosis as revealed by Sirius Red staining (Figure 8C,D). After 2 weeks of recovery, these cell populations returned to baseline, and fibrosis largely resolved. In contrast, *NIK* ^*Tg*^ mice exhibited markedly greater expansion of cholangiocytes, macrophages/Kupffer cells, and HSCs, accompanied by more severe fibrosis (Figure 8D). Notably, unlike *NIK* ^*Con*^ mice, *NIK* ^*Tg*^ mice failed to recover after TAA withdrawal, with persistent accumulation of these cell types and sustained liver fibrosis. In line with these results, liver α‐SMA protein levels were more strongly induced by TAA in *NIK* ^*Tg*^ mice than in *NIK* ^*Con*^ mice and remained elevated even after TAA withdrawal (Figures 8E and S4). Taken together, these results indicate that aberrant biliary NIK activation hinders liver injury resolution and fibrosis regression, thereby contributing to poor prognosis.

## 4 Discussion

Upregulation of biliary NIK is commonly observed in humans and rodents with chronic liver disease [4]. This study aimed to investigate whether and how aberrant NIK activation promotes liver disease progression, using both cholangiocyte‐specific *NIK* transgenic mice and *NIK*‐null cholangiocyte cultures. Under basal conditions, *NIK* ^*Tg*^ mice appeared grossly normal, indicating that the *NIK* transgene alone is insufficient to cause liver injury. However, upon exposure to TAA or DDC, *NIK* ^*Tg*^ mice displayed dramatically increased mortality and markedly exacerbated liver pathology compared to wild‐type *NIK* ^*Con*^ littermates. This included a pronounced ductular reaction, heightened liver inflammation, and extensive fibrosis. These findings suggest that aberrant biliary NIK activation significantly heightens liver susceptibility to toxic insults, positioning it as a critical driver of liver disease progression.

In both in vivo and in vitro models, we found that NIK directly promotes cholangiocyte proliferation while inhibiting cholangiocyte death. These effects likely contribute to cholangiocyte expansion and the ductular reaction observed during liver disease progression. Reintroduction of NIK into HuCC‐T1^NIK−/−^ cholangiocyte culture fully rescued both the proliferative and anti‐cell death phenotypes. In contrast, the NIK^G885R^ mutant—deficient in activating the noncanonical IKKα/NF‐κB2 pathway [18, 19]―exhibited a significantly reduced capability to regulate cholangiocyte proliferation and survival. These results suggest that biliary NIK promotes cholangiocyte expansion and ductular reaction through both IKKα/NF‐κB2‐dependent and ‐independent mechanisms.

In addition to regulating cholangiocyte growth and expansion, we found that biliary NIK also influences the crosstalk of cholangiocytes with immune cells and HSCs, thereby critically shaping the liver microenvironment. Deletion of *NIK* in HuCC‐T1^NIK−/−^ cholangiocytes significantly decreased the secretion of cholangiokines that activate BMDMs and LX2 cells (a model of HSCs). Re‐expression of NIK in these cells fully rescued cholangiokine secretion. Importantly, both NIK^ΔC287^ and NIK^G885^―both deficient in activating the noncanonical IKKα/NF‐κB2 pathway—displayed a markedly impaired ability to increase cholangiokine secretion. These results suggest that biliary NIK promotes cholangiokine secretion by both IKKα/NF‐κB2‐dependent and ‐independent mechanisms. Collectively, our results unveil a previously unrecognized biliary NIK‐cholangiokine‐macrophage/Kupffer cell‐HSC axis that drives the development of immune/fibrotic microenvironments. Furthermore, we provided proof‐of‐concept evidence that this NIK‐elicited pathogenic niche plays a crucial role in orchestrating liver disease progression.

In liver disease treatment, achieving liver injury resolution and fibrosis regression remains a major challenge. Surprisingly, we found that *NIK* ^*Tg*^ mice, unlike *NIK* ^*Con*^ littermates, failed to recover from TAA‐induced liver injury and fibrosis after cessation of toxin exposure. These findings demonstrate that aberrant activation of biliary NIK is sufficient to sustain liver injury and fibrosis. We propose that NIK‐induced pathogenic cholangiokines persistently stimulate live immune cells and HSCs, thereby preventing injury resolution. Importantly, aberrant activation of hepatic NIK blocks regenerative hepatocyte proliferation while exacerbating hepatic oxidative stress and damage [16, 26]. It is therefore highly likely that biliary and hepatic NIK pathways act synergistically to halt liver injury resolution, fibrosis regression, and liver regeneration. These findings underscore the importance of aberrant activation of liver NIK in driving poor prognosis and highlight the therapeutic potential of NIK inhibition as a strategy for liver disease treatment.

Several caveats should be considered in interpreting our findings. The composition of cholangiocyte‐conditioned medium is complex and likely contains additional molecules beyond the putative cholangiokines, which may influence the observed phenotypes and complicate data interpretation. Moreover, the specific identities of these cholangiokines remain unknown. Future studies are needed to characterize these factors and clarify their individual contributions.

## Author Contributions

Y.W. and L.R. conceived and designed the experiments. Y.W. performed the experiments. R.N. and Q.K. generated HuCC‐T1^NIK−/−^ cells. Y.W. and L.R. wrote the manuscript. Y.W., R.N., Q.K., and L.R. edited the manuscript.

## Funding

This study was supported by grants R01 DK114220, R01 DK130111, and R01 DK141559 (L.R.) from the National Institutes of Health. This work utilized the cores supported by the Michigan Diabetes Research and Training Center (NIH DK020572), Michigan Metabolomics and Obesity Center (DK089503), and the University of Michigan Center for Gastrointestinal Research (NIDDK P30DK034933).

## Conflicts of Interest

The authors declare no conflicts of interest.

## Supporting information


**Data S1:** fsb271318‐sup‐0001‐supinfo.pdf.

## Data Availability

All data supporting the findings described in this paper is available in the article and in the Supporting Information.
