# Supplementary material for: Biliary NF‐κB‐Inducing Kinase Promotes Ductular Reaction, Inflammation, and Fibrosis and Impedes Liver Disease Resolution
Source: FASEB J. 2025 Dec 5;39(23):e71318. doi: 10.1096/fj.202502014RRR (PMC12680040; doi:10.1096/fj.202502014RRR)
Supplement: Supplementary file 1 — Data S1: fsb271318‐sup‐0001‐supinfo.pdf. [file FSB2-39-e71318-s001.pdf]

Supplemental Information

Biliary NF-κB-inducing kinase promotes ductular reaction, inflammation, and fibrosis and impedes liver disease resolution

Yina Wang, Rohollah Nikooie, Qianqian Kang, Liangyou Rui

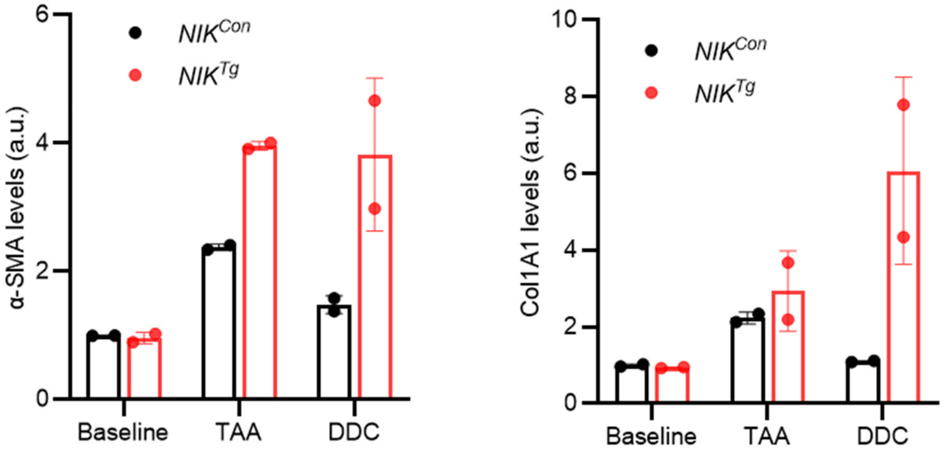

Supplemental Figure S1. Biliary NIK enhances liver fibrosis. *NIK*^{Tg} and *NIK*^{Con} male mice were treated with TAA or DDC for 4 weeks. Liver extracts were immunoblotted with anti-α-SMA and anti-Col1A1 antibodies (Figure 4E). α-SMA and Col1A1 levels were quantified and normalized to β-actin levels (n=2). Data are presented as mean ± SD.

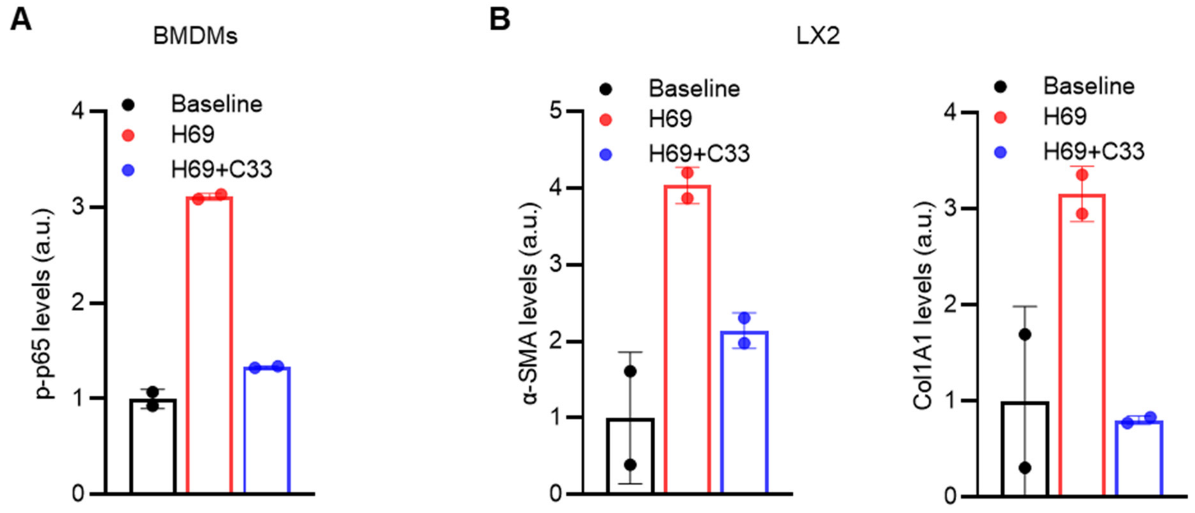

Supplemental Figure S2. NIK promotes cholangiokine secretion. (A) BMDMs were treated with the indicated CMs for 24 h, and cell extracts were immunoblotted with the indicated antibodies (the same blots as in Figure 5B). p65 phosphorylation was quantified and normalized to total p65 levels (n=2). (B) LX2 cells were treated with the indicated CMs for 24 h, and cell extracts were

immunoblotted with the indicated antibodies (the same blots as in Figure 5D). α-SMA and Col1A1 levels were quantified and normalized to β-actin levels (n=2). Data are presented as mean ± SD.

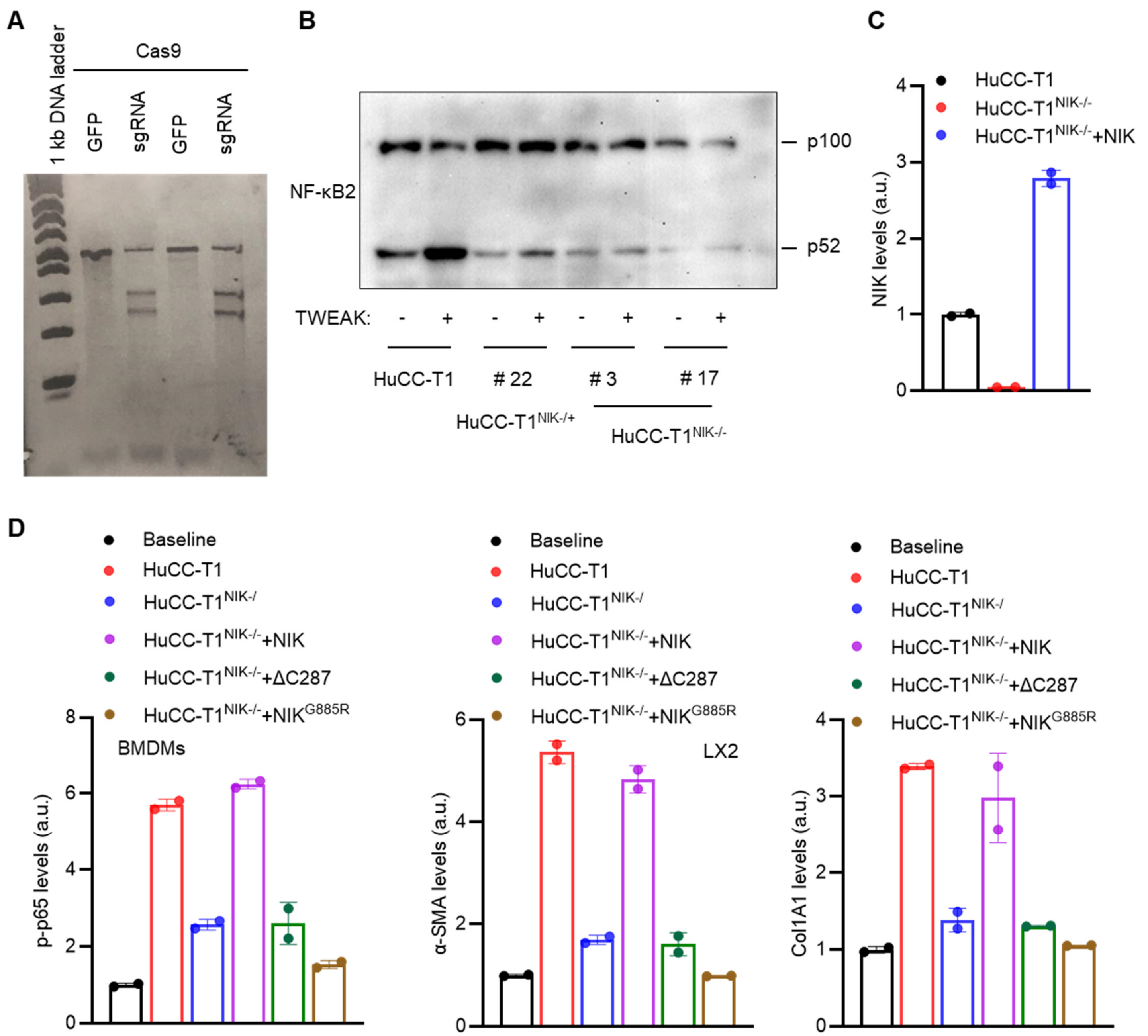

Supplemental Figure S3. Generation of HuCC-T1$^{NIK-/-}$ cells using CRISPR/Cas9. (A) Genomic DNA was isolated from HuCC-T1 cells 3 days post transfection with plasmids expressing GFP (control) or sgRNA. *MAP3K14/NIK* gene editing was revealed by T7 endonuclease I (T7E1)-mediated cleavages using a T7E1 assay. (B) HuCC-T1 and HuCC-T1$^{NIK-/-}$ cells were treated with TWEAK (10 ng/ml) for 10 h. Cell extracts were immunoblotted with anti-NF-κB2 antibody. Clones #3 and #17 were homozygous, while #22 was heterozygous. (C) HuCC-T1$^{NIK-/-}$ cells were transfected with NIK plasmids, and cell extracts were immunoblotted with anti-NIK antibody (Figure 5E). NIK levels were

quantified and normalized to β-actin levels (n=2). (D) BMDMs and LX2 cells were treated with the indicated CMs for 24 h, and cell extracts were immunoblotted with the indicated antibodies (Figure 7C-D). p65 phosphorylation was quantified and normalized to total p65 levels; α-SMA and Col1A1 levels were normalized to β-actin levels (n=2).

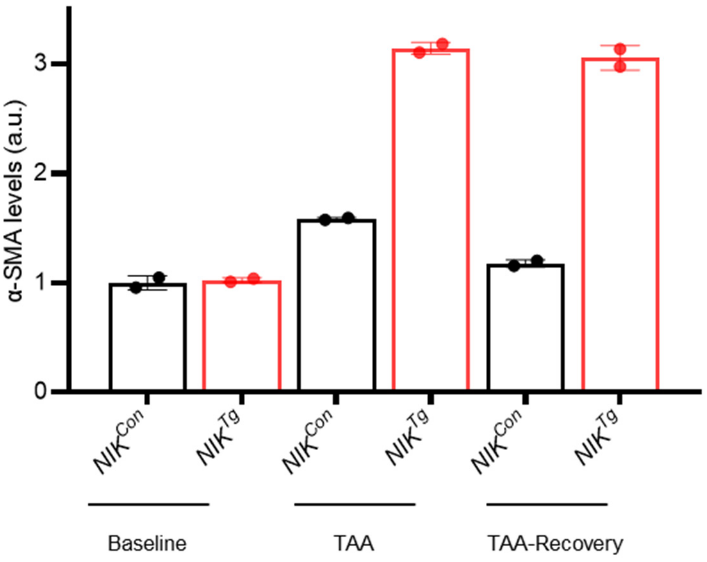

Supplemental Figure S4. NIK hinders liver injury resolution and fibrosis regression. *NIK*^{Tg} and *NIK*^{Con} male mice were treated with TAA or DDC for 4 weeks followed by 2 weeks of recovery. Liver extracts were immunoblotted with anti-α-SMA antibody (Figure 8E). α-SMA levels were quantified and normalized to β-actin levels (n=2). Data are presented as mean ± SD.

ANTIBODY	SOURCE	Cat#	Blot	IHC
α-SMA	Cell Signaling Technology	19245S, RRID: AB_2734735	1:1000	1:200
Col1A1	Cell Signaling Technology	72026S, RRID: RRID: AB_2904565	1:1000	
phospho-p65	Cell Signaling Technology	3033S, RRID: AB_331284	1:1000	
p65	Cell Signaling Technology	8242S, RRID: AB_10859369	1:1000	
NIK	Abcam	ab203568, RRID:	1:1000	
phospho-ERK	Cell Signaling Technology	4370L, RRID: AB_2315112	1:1000	
ERK	Cell Signaling Technology	4695S, RRID: AB_390779	1:1000	

β-actin	Abclonal Techonology	AC026, RRID: RRID: AB_2768234	1:1000	
HA-Tag	Thermo Scientific	26183, RRID: RRID: AB_10978021	1:1000	
anti-Mouse IgG	Abcam	ab6728, RRID: AB_955440	1:5000	
anti-Rabbit IgG	Abcam	ab6721, RRID: RRID: AB_955447	1:5000	
CK19	DSHB Antibodies	TROMA-III, RRID: AB_2133570		1:100
Ki67	Cell Signaling Technology	12202S, RRID: AB_2620142		1:200
Cleaved Caspase-3	Cell Signaling Technology	9664S, RRID: AB_2070042		1:100
MPO	Abclonal Techonology	A1374, RRID:		1:200
anti-Rabbit IgG AF555	Cell Signaling Technology	4413S, RRID: AB_10694110		1:1000
anti-Rabbit IgG AF488	Cell Signaling Technology	4412S, RRID: AB_10694110		1:1000
anti-Rat IgG AF555	Cell Signaling Technology	4417S, RRID: AB_10694111		1:1000
anti-Rat IgG AF488	Cell Signaling Technology	4416S, RRID: AB_10693769		1:1000

Supplemental Table 1. Antibody list.

**Genes**	**Forward**	**Reverse**
18S	CGCTTCCTTACCTGGTTGAT	GAGCGACCAAAGGAACCATA
Col1a1	TCACCTACAGCACCCTTGTG	GGTGGAGGGAGTTTACACGA
IL-1β	GCCTTGGGCCTCAAAGGAAAGAATC	GGAAGACACAGATTCCATGGTGAAG
IL-6	*AGCCAGAGTCCTTCAGA*	*GGTCCTTAGCCACTCCT*
Timp1	*GCTAAATTCATGGGTTCCCCAG*	*GAGAAAGCTCTTTGCTGAGCAG*
TNFα	*CATCTTCTCAAAATTCGAGT GACAA*	*TGGGAGTAGACAAGGTACAACCC*
Vimentin	*GACCTCACTGCTGCCCTGCG*	*GACTCCTGCTTGGCCTGGCG*
α-SMA	*GTTCAGTGGTGCCTCTGTCA*	*ACTGGGACGACATGGAAAAG*
iNos	CAGGGCCACCTCTACATTTG	TGCCCCATAGGAAAAGACTG
GAPDH (h)	TGCACCACCAACTGCTTAGC	ACAGTCTTCTGGGTGGCAGTG
α-SMA (h)	AAAAGACAGCTACGTGGGTGA	GCCATGTTCTATCGGGTACTTC
Col1a1 (h)	AACATGACCAAAAACCCAAAGTC	CATTGTTTCCTGTGTCTTCTGG
Timp1 (h)	GGGCATGGATAAACAGGGAAACA	ACGCTGGTATAAGGTGGTCTG
Vimentin (h)	GGACCAGCTAACCAACGACA	AACCCGACGCAGAGGATTTTCT

Supplemental Table 2. Primer list.
